# Buying Organic Food Products: The Role of Trust in the Theory of Planned Behavior

**DOI:** 10.3389/fpsyg.2020.575820

**Published:** 2020-10-23

**Authors:** Luigina Canova, Andrea Bobbio, Anna Maria Manganelli

**Affiliations:** Department of Philosophy, Sociology, Education and Applied Psychology, University of Padua, Padua, Italy

**Keywords:** Theory of Planned Behavior, organic food products, organic fruit and vegetables, trust, two-wave study, structural equation modeling

## Abstract

When someone decides to buy organic food products trust plays a role. Consumers, in fact, are neither supposed to have the appropriate knowledge to evaluate the characteristics of these products, nor can they control that the food was actually manufactured following the procedures prescribed by organic production. Therefore, trust may contribute to the explanation of both purchasing intention and behavior since it represents a heuristic or shortcut that people adopt in order to reduce the large amount of information that consumers need to take into account. The present research aimed to analyze the role of trust in organic products on buying behavior adopting the Theory of Planned Behavior (TPB) as theoretical framework. A relational model was tested in which this variable was supposed to act as a background factor associated with all the classical constructs foreseen by the theory and the buying behavior. Also, indirect effects of trust on both intention and behavior were assessed. Two studies were conducted targeting the purchase of organic food products in general (Study 1) and of fresh organic fruit and vegetables (Study 2). In both studies, the data collection was organized in two waves, with a time lag of 1 month. At Time 1, the questionnaires included measures of intention, its antecedents and trust, while at Time 2 self-reported buying behavior was collected. Data were supplied by two convenience samples of Italian adults (237 and 227 participants) and analyzed via structural equation modeling. Results turned out to be overlapping in both studies, since trust was positively associated with attitude and subjective norm, and it was indirectly associated with intention and behavior, thanks to the mediation of the TPB constructs. The outcomes highlighted the importance of people’s trust in organic products as a meaningful antecedent that boosts the TPB-based psychosocial processes that are supposed to stand behind both purchasing intentions and behaviors.

## Introduction

Sustainable consumption in the food sector is one of the main strategies for achieving environmental sustainability. The most effective ways to reduce the environmental impact of food consumption from the consumer perspective are the refusal of air-transported food, the preference for organic food, and the reduction in meat consumption (Jungbluth et al., 2000). Organic production is defined as a complex and intertwined system of both farm management and the food production chain that aims to merge best environmental practice, a high level of biodiversity, the conservation of natural resources, high animal welfare standards, and a production method employing natural constituents and processes, as an example free from synthetic chemical substances and genetically modified organisms (European Commission, 2014). A major challenge in this sector is to both expand and respond to demand without compromising consumers’ confidence in the above-mentioned principles and processes, as well as to build trust in the organic products imported, particularly as regards control measures (European Commission, 2014). Trust, credibility, transparency, and safety are key aspects of this sector, as ways of ensuring overall benefits in the long-term perspective (European Commission, 2016).

Organic agriculture has developed rapidly in Europe and North America in response to the feedback coming from both markets and in terms of consumers’ demands. In Europe, as an example, the organic market has continued to grow (Willer et al., 2020a), and data from FiBL-AMI Survey (Willer et al., 2020c) showed that between 2000 and 2018 the retail sales of organic food has reached more than 40 billion euros. The largest European market for organic food in 2018 was Germany, with retail sales of 10.9 billion euros, followed by France (9.1 billion euros), and Italy (3.5 billion euros) (Willer et al., 2020b). According to the Bioreport 2017–2018 (Viganò, 2019), in 2017 the value of sales for the domestic use of organic food and drink in Italy grew by 18.6%, compared to in 2016. In 2018 the purchase of organic food products represented 3.7% of all food purchases, while in 2000 it was 0.7% (Nomisma, 2018). Furthermore, between June 2018 and June 2019 there was an increase in sales of organic products for domestic use in large retailers of 6% (Nomisma, 2019). Between March 2019 and March 2020, sales of organic fruit and vegetables recorded a growth of 24.8% (Assobio, 2020).

In the last two decades, research on sustainable food consumption has increased (Scalco et al., 2017), with contributions coming from scholars belonging to different fields. This trend reflects both the interdisciplinary nature of this research field and the interest shown—among others—by economists, nutritionists, and social psychologists. Although most of the research has been carried out in the United States and in Europe (Italy included), a growing interest has also emerged in recent years among scholars from other geographical areas, such as the Far East, Iran, China, and India (e.g., Teng and Wang, 2015; Yazdanpanah and Forouzani, 2015; Yadav and Pathak, 2016a, b, 2017; Nuttavuthisit and Thøgersen, 2017; Qi and Ploeger, 2019).

Many studies within the food consumption literature have assumed the Theory of Planned Behavior (TPB) (Ajzen, 1991) as their theoretical reference for investigating the psychosocial factors that explain consumers’ intentions and behaviors. In brief, the TPB postulates that a given behavior is determined by the intention to execute it. Intention captures both motivations and cognitive planning, and it is an immediate antecedent of the behavior itself. Intention is a function of three factors, which are also related to each other: attitude toward the behavior, subjective norm, and perceived behavioral control (PBC). PBC can predict the behavior both directly and indirectly, thanks to the mediation of intention. The TPB has been applied successfully in a wide range of fields, such as those concerning health behaviors (McEachan et al., 2016), healthy eating (Riebl et al., 2015), pro-environmental behaviors (Klöckner, 2013), and organic food consumption (Scalco et al., 2017), and its predictive power has been demonstrated in a number of meta-analyses like those just mentioned.

**FIGURE 1:**
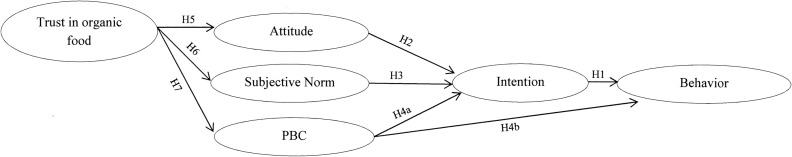
Hypothesized model. PBC, Perceived Behavioral Control.

In the present study, we investigated the role of consumer trust considered as a background factor within an extended TPB model. Figure 1 shows the conceptual model. The paper presents two studies based on a prospective design and with data collected in two waves (Time 1 and Time 2, 1 month later). The first study considered the purchase of organic food in general as target behavior; the second was focused on the purchase of organic fruit and vegetables. On the one hand, we expect the first study to contribute to the understanding of the psychological processes behind the purchase of organic foods in general: indeed, “organic food” is intended to be a label or brand, which can nowadays be applied to a wide range of products. On the other hand, in the second study, fruit and vegetables were chosen because they represent, since 2000, the largest portion of the Italian organic food market, and the demand for them is growing rapidly (Saba and Messina, 2003; Ricci et al., 2018). In fact, the percentage of Italian families that had bought organic fruit and vegetables at least once in the last year increased from 53% in 2012 to 81% in 2018 (Nomisma, 2018). In particular, sales of organic fruit in 2017 were 12.3% higher compared to in 2016 (Viganò, 2019).

### Aims and Hypotheses

Following the TPB, the first aim of the studies was to offer a contribution to the prediction and explanation of the intention to purchase organic food (in general or fruit and vegetables) at Time 1, and of self-reported behavior at Time 2. A second aim was to investigate how consumer trust is related to both intention and behavior. Finally, thanks to the analysis of the results from Studies 1 and 2, a third aim was to explore the possible similarity of the processes leading to purchasing intentions and to actual purchasing behaviors in the case of a general versus a specific target.

Many TPB-based studies use a prospective design and measure behavioral responses weeks or months after having measured attitudes, subjective norms, PBC, and intentions (Fishbein and Ajzen, 2010). Instead, in the case of green purchasing behavior, research tends to focus only on intention, while the effect on actual behavior is only assumed. For example, the recent meta-analysis by Scalco et al. (2017), based on twenty-three studies, revealed that the majority of them did not report the relationships between intentions and behaviors while only six reported the relationships between intentions and past or current behaviors. In any case, Scalco et al. (2017) found that the correlations between intentions and actual behaviors ranged between moderate and large. More recently, as far as we know, only one study on the purchase of organic milk (Carfora et al., 2019) considered future behavior and attested the predictive role of intention.

Consequently, as assumed by the TPB and as shown by previous evidence referred to the TPB framework, we proposed the subsequent hypothesis:

H1. Intentions will predict self-reported future purchasing behavior of organic food in general, or organic fruit and vegetables in particular.

Attitudes toward a behavior express individuals’ global positive/negative evaluations of it; they predict intentions and, consequently, behaviors. Aertsens et al. (2009) stated in their review that numerous studies on organic food consumption reported a positive and significant relationship between the attitude toward buying this kind of food and the intention to buy it—something that is consistent with the TPB. Therefore, also in the context of organic food consumption, attitude appears to play a crucial role in shaping behavior by its direct association with intention (Scalco et al., 2017). So, based on the TPB and previous results, we posed the following hypothesis:

H2. There will be a positive relationship between consumers’ attitudes toward the purchase of organic food in general, or organic fruit and vegetables in particular, and their intentions to purchase them.

Subjective norm, the second antecedent of intention, is an expression of normative influence. It reflects people’s perception of what the most important referent individuals or groups, especially family and friends, consider to be an acceptable or unacceptable behavior. The effectiveness of subjective norm in explaining intention and behavior is debated in the literature, and results are mixed. Armitage and Conner (2001) argued that the normative component of the TPB might represent the comparatively weaker construct of the TPB. Nonetheless, the meta-analysis by Scalco et al. (2017) demonstrated the significant role played by subjective norm in shaping the intention to buy organic food products. Consequently, we hypothesize as follows:

H3. Subjective norms will be positively associated with intentions to purchase organic food in general, or organic fruit and vegetables in particular.

PBC refers to people’s perceptions of the easiness or difficulty of performing the behavior of interest (Ajzen, 1991), and it is considered to be a suitable proxy for actual control (Fishbein and Ajzen, 2010). PBC contributes to the prediction of both intention and behavior. In the case of complete control over behavior, PBC is an antecedent of intention, and intention alone predicts behavior; when the behavior is not completely under the person’s volitional control, it may predict behavior directly. The strength of the association between PBC and intention varies across studies. In some cases, PBC had a significant impact on the intention to buy organic food (e.g., Zagata, 2012; Maichum et al., 2016; Yadav and Pathak, 2016a, b; Carfora et al., 2019; Wang et al., 2019; Fleşeriu et al., 2020); in others, the effect was not significant (e.g., Al-Swidi et al., 2014; Yazdanpanah and Forouzani, 2015). In the meta-analysis by Scalco et al. (2017), PBC seemed to play a minor role compared to attitude and subjective norm with respect to intention prediction. These different findings can be attributed to both the degree of availability of organic food in different contexts and to several dissimilarities regarding the items used to measure this construct. However, our hypotheses were as follows:

H4a. PBC will be positively associated with intentions to purchase organic food in general, or organic fruit and vegetables in particular.

H4b. PBC will be positively associated with future purchase of organic food in general, or organic fruit and vegetables in particular.

The TPB allows many background factors (e.g., age, sex, ethnicity, socioeconomic status, education, personality, past experiences) to act as sources of potential influence on the beliefs people hold (de Leeuw et al., 2015; Hagger and Hamilton, 2020). In the TPB integrated model developed for this study (Figure 1), we considered trust in organic food as a background variable. In fact, trust is a behavioral determinant whose nature may be relevant for all the TPB constructs: attitude, subjective norm and PBC (Mazzocchi et al., 2008).

In the organic food market, consumer trust is a crucial issue. Most consumers do not have the expertise, knowledge, and other resources to properly understand the characteristics distinguishing organic food, and so organic is a sort of credence quality (Nuttavuthisit and Thøgersen, 2017). Furthermore, not even after consumption the consumer can verify whether a product is organic and therefore trust in the product’s integrity is an essential driver for the consumer to buy it. The lack of consumer trust in green products can act as a barrier to green consumption (Joshi and Rahman, 2015); vice versa, uncritical trust in the “organic food” category or label may leave consumers at the mercy of marketers. Consequently, it is necessary to investigate more thoroughly the role of consumer confidence as regards organic food and to analyze the strength of its influence on intention and purchase behavior.

In the literature, it is possible to find several definitions of trust. One that is useful for our aims is that of Hobbs and Goddard (2015), which defines trust as “a heuristic that might be used in situations where lack of knowledge, experience or familiarity with firms, products or process used to create products hampers decision making” (p. 72). Trust has also been viewed as “a state of perceived vulnerability or risk that is derived from individual uncertainty regarding motives, intentions, and potential actions of others on whom they depend” (Kramer, 1999, p. 571). This last definition captures two important dimensions of the concept of trust: (a) the expectation that the counterpart will act in a reliable and not harmful manner and (b) the intention to rely on the counterpart, at the same time as accepting some degree of vulnerability (e.g., uncertainty, risk of being frustrated) (Singh and Sirdeshmukh, 2000).

Some studies considered trust as an additional predictor of the intention to buy eco-friendly food (e.g., Menozzi et al., 2015; Giampietri et al., 2018; Carfora et al., 2019) and found that trust was a significant antecedent of intention, explaining additional quotas of intention variance with respect to the classical TPB constructs. Very few studies tested the extent to which relationships between trust and intention and between trust and behavior were mediated by TPB constructs.

The proposed mediation of relations between trust, intention and behavior by TPB constructs can be summarized in the model presented in Figure 1 where trust is proposed as predictor of attitude, subjective norm and PBC.

Previous studies confirmed that trust is an important predictor of customer attitudes and when the TPB was assumed as the theoretical framework, trust was identified as an antecedent of attitudes toward purchasing behavior (Teng and Wang, 2015; Ricci et al., 2018). To the best of our knowledge, no study in the field of organic food choice has analyzed the relationships between trust, subjective norm, and PBC, but some studies in the transport literature (e.g., Hsiao and Yang, 2010; Madha et al., 2016; Borhan et al., 2017; Ibrahim et al., 2020) and on on-line transactions (e.g., Wu and Chen, 2005) highlighted that trust via attitude, subjective norm and PBC had positive and indirect relationships with behavioral intention. Capitalizing on these results, our hypothesis concerning the link between trust and attitude was the following:

H5. Trust in organic food will be positively associated with the attitude toward the purchase of organic food in general or organic fruit and vegetables in particular.

According to the TPB, subjective norms refer to people’s perceptions of important referents’ beliefs about the behavior. The positive association between individual trust and subjective norm means that those who have a higher degree of trust in purchasing organic food should rely more on their referent beliefs. This confidence in significant others and their beliefs can be expected to play a role in determining the subjective norms; in fact individuals will be more willing to comply with the important referents (Wu and Chen, 2005). So our hypothesis here was as follows:

H6. Consumers’ trust in the purchase behavior of organic food in general, or organic fruit and vegetables in particular, will be positively associated with subjective norm

In regards to PBC, trust can act as a resource that aids consumers to gain control over purchase through self-efficacy. Self-efficacy is built through self-confidence and mutual trust in interpersonal relationships; hence, trust between consumers and sellers or producers of organic food that behave in accordance with consumers’ expectation should increase consumers self-efficacy and, in turn, increase PBC (Wu and Chen, 2005). So we developed the following hypothesis:

H7. Consumers’ trust in organic food will be positively associated with perceived behavioral control.

Finally, the three antecedents of intention (attitude, subjective norm, and PBC) are supposed to mediate the relationship between trust and intention, and the three antecedents of intention along with intention itself will mediate the relationship between trust and purchase behavior. Therefore, we hypothesized the following:

H8. Trust will be positively and indirectly associated with purchase intentions of organic food in general, or organic fruit and vegetables in particular, via attitudes, subjective norms, and PBC.

H9. Trust in organic food will be positively and indirectly associated with the future purchase behavior of organic food in general, or organic fruit and vegetables in particular, via attitudes, subjective norms, PBC, and intentions.

In conclusion, our study aimed to apply the TPB model, extended with measures of trust in organic food, and to offer an original contribution to the issue of predicting both the intention to buy and the purchasing behavior of organic food in general (Study 1) and organic fruit and vegetables in particular (Study 2).

Compared to the extant literature, we believe that our study can be considered to be innovative for three reasons. First, in both studies, self-reported actual purchases were assessed, while most research has limited the analysis to the intention to purchase. Second, data were collected in two waves, thus offering the possibility to separate the background measures (such as trust) and the measures of all the classical TPB constructs from the target measures, offering the chance to assess the predictive power of the hypothesized model. Third, the role of trust was questioned, given its possible heuristic role within social-cognitive processes existing behind both intention formation and behavioral execution in the case of organic food in general or organic fruit and vegetables in particular. Finally, the qualitative comparison between the results of Studies 1 and 2 can be seen as promising in order to sketch some general conclusions concerning the possible similarities of the TPB-based processes in the two different conditions (general *vs.* specific types of products). This will offer suggestions for both scholars and practitioners interested in the interplay between cognitive and behavioral processes related to the field of organic food choice and consumption.

## Materials and Methods

### Procedure and Participants

For both studies, the data collection was organized in two waves, Time 1 and Time 2. At Time 1, participants completed a structured anonymous questionnaire including the measures of the extended TPB model and socio-demographic variables. At Time 2, 1 month later, participants’ self-reported behavior measures were collected. Since the two studies followed exactly the same design, the following description applies to both.

For each study, about one hundred university students from two different courses offered by the School of Psychology at Padua University were engaged in data collection. Students were asked to administer the questionnaire among three or four of their friends, relatives, or acquaintances who did not belong to the same family. Participants were provided with an envelope containing the questionnaire, an instruction letter, and an informed consent form that participants had to sign and return before completing the questionnaire at Time 1. In the instruction letter, participants were informed about the aim of the study, the purchasing behavior the study was focused on (i.e., the purchase of organic food or the purchase of organic fruit and vegetables), the estimated duration of the task, and the possibility of withholding their consent to participate at any time, and they were also assured that all answers would remain confidential. Each participant filled in the questionnaire autonomously and gave it back immediately. Informed consent forms and completed questionnaires were collected using separate envelopes and returned to the researchers by the students. One month later (Time 2), through scheduled appointments, the participants filled in the second questionnaire and were quickly debriefed.

In Study 1, there were 400 potential participants, but usable data was obtained from 371 individuals (response rate: 92.7%). Among them, 288 completed the second questionnaire (final response rate: 72%). Finally, participants who declared that they were not at least partially responsible for purchasing decisions regarding food products were excluded, so the final sample comprised 237 participants.

In Study 2, 300 potential participants were contacted, and usable data was obtained from 260 individuals (response rate: 86.7%). Among them, 233 also completed the second questionnaire (final response rate: 77.7%). The same exclusion criterion as in Study 1 was applied, so the final sample comprised 227 participants. Table 1 provides the socio-demographic composition of the two samples.

**TABLE 1:** Survey sample characteristics.

	Study 1 (*n* = 237)		Study 2 (*n* = 227)	
Demographics				
Age	19–70 years	*M* = 36.49, *SD* = 14.36	18–75 years	*M* = 39.58, *SD* = 15.45

	**N**	**%**	**N**	**%**

**Gender**				
Women	154	65	148	65.2
Men	82	34.6	79	34.8
Missing data	1	0.4	0	0
**Italian geographic area**				
Northeast	193	81.4	93	41
Northwest	22	9.3	10	4.4
Central	8	3.4	10	4.4
Southern	13	5.5	112	49.3
Missing data	1	0.4	2	0.9
**Occupation**				
Employed	125	52.7	117	51.5
Out of work (housewife, students, retired, unemployed)	110	46.4	106	46.7
Missing data	2	0.8	4	1.8
**Education**				
Compulsory school	31	13.1	26	11.4
High school	119	50.2	142	62.6
University degree	86	36.3	56	24.7
Missing data	1	0.4	3	1.3
**Marital status**				
Married or cohabiting	94	39.7	91	40.1
Single	143	60.3	136	59.8
**Parental status**				
Dependent children	81	34.2	93	41
No dependent children	156	65.8	134	59
Family net monthly income (in euros)				
Below 1,500	43	18.1	64	28.2
1,501–2,500	88	37.1	60	26.4
2,501 and above	98	41.4	91	40.1
Missing data	8	3.4	12	5.3
**Where do you buy organic products?**				
Large retailers	173	73	185	81.5
Small retailers	121	51.1	124	54.6
Direct manufacturer	86	36.3	120	52.9
Street markets	69	32.7	91	40.3
E-commerce	7	3.3	24	10.6

A drop out analysis indicated in the case of Study 1 only one difference between the 237 participants included in the final sample and the 73 “drop outs” (participants who did not filled in the second questionnaire). In the final sample vs. “drop outs” there were more respondents that declared to be single than married or cohabiting (χ^2^_1_ = 5.21, *p* < 0.03). No difference was found regarding TPB constructs and trust. As regards Study 2, the same analysis showed very few differences between the 227 participants included in the final sample and the 23 “drop outs.” The former group scored significantly lower on both PBC (M_*final sample*_ = 4.55 vs. M_*drop out*__*s*_ = 5.65, *t*_248_ = −3.29, *p* < 0.002) and intention (M_*final sample*_ = 4.37 vs. M_*drop out*__*s*_ = 5.20, *t*_248_ = −2.22, *p* < 0.03) than the latter.

Overall, even if the people in Study 1 were slightly unbalanced in terms of where they lived in Italy compared to those of Study 2, we concluded that the typical participant was predominantly a middle-aged woman, currently in the workforce, and with at least a high-school education. The majority declared that they were single, did not have children, and were earning a salary, which was, according to national statistics, above the average level (ISTAT, 2019). As regards their supply sources of organic products, most respondents used large and small retailers, as well as direct manufacturers. This evidence supports the expanded availability of organic products, which is connected with the increase in consumer demand that has been experienced in the last decades.

### Measures

The questionnaires presented measures of TPB constructs adapted from those already used in previous studies in the Italian context (Canova and Manganelli, 2016; Canova et al., 2020). The measures complied with the TPB questionnaire construction guidelines (Fishbein and Ajzen, 2010). In Study 1, the target behavior was the purchase of organic food products in the following month. For all participants, the following description of target behavior was provided: “The purchase of organic products, i.e., products coming from organic farming (e.g., cereals, fresh fruit and vegetables, honey and jams, milk and derivatives, oil, bread, tomato sauce and seasonings, wine, dried fruit, pickles, meat and fish), from any point of sale (e.g., supermarkets, specialized stores, small shops, hard discount shops, fair-trade shops, local markets) in the next month.” In Study 2, the target behavior was the purchase of fresh organic fruit and vegetables in the next month. For all participants, the following description of target behavior was provided: “The purchase of fresh organic fruit and vegetables from any point of sale (e.g., supermarkets, specialized stores, small shops, hard discount shops, fair-trade shops, local markets) in the next month.”

During the first wave of the studies, participants were asked to report their attitude, subjective norm, PBC, trust in organic food, and demographic information.

#### Trust in Organic Food

Three items derived from the literature were adopted (Giampietri et al., 2018): “I perceive organic food to be reliable,” “I trust in organic food products,” and “I trust in purchasing organic food products.” The response scale ranged from 1 (strongly disagree) to 7 (strongly agree).

#### Attitude Toward the Behavior

Attitude was measured by presenting the participants with the statement: “To buy organic food products/organic fruit and vegetables in the next month would be…” and asking them to respond on four 7-point semantic differential adjective scales (unpleasant–pleasant, useless–useful, negative–positive, and crazy–wise). The response scales were anchored from 1 (negative pole) to 7 (positive pole).

#### Subjective Norm

The participants were asked to respond on a 7-point Likert-type scale to two items: “Most of the people who are important to me (family, friends, acquaintances, partners) think I should/should not buy organic food products/organic fruit and vegetables in the next month” and “Most people who are important to me would like me to buy organic food products/organic fruit and vegetables in the next month.” The anchors varied for each question from 1 (I should not/false) to 7 (I should/true).

#### Perceived Behavioral Control

This was measured with two items: “To what extent do you think buying organic food products/organic fruit and vegetables in the next month is a behavior under your control?” and “How much control do you think you have over buying organic food products/organic fruit and vegetables in the next month?” The anchors varied for each question from 1 (Not at all/no control) to 7 (Very much/complete control).

#### Intention

Three items were used: “I intend to buy organic food products/organic fruit and vegetables in the next month,” “How likely is it that you will form the intention to buy organic food products/organic fruit and vegetables in the next month?” and “How likely is it that you will actually buy organic food products/organic fruit and vegetables in the next month?” Lower points on the response scale (i.e., 1) indicated both low agreement and likelihood, whereas higher points (i.e., 7) indicated high agreement and likelihood.

#### Demographics

Gender, age, marital and parental status, the geographic area of residence, employment status, education, and net (i.e., after taxation) monthly income were assessed, along with where they predominantly purchased organic products (e.g., large retailers, small retailers, direct manufacturers and solidarity purchase groups, street markets). Moreover, the participants were asked: “Are you responsible for making decisions regarding the buying of food products?” (1 = Yes, I am the main person responsible for making decisions about buying food products; 2 = I am one of the people responsible for making decisions about buying food products; and 3 = No, I am not involved in the decision-making process about buying food products).

#### Self-Reported Behaviors

At Time 2, the participants had to report their buying behavior with reference to the month immediately before the second wave of the research. For this purpose, we used two items. In both studies, the first item was: “In the last month, have you personally bought organic food/organic fruit and vegetables?” with a response scale ranging from 0 (No, never) to 4 (Yes, regularly, every time I went shopping). The second item was different in each of the two studies; in Study 1, it was as follows: “In the last month, how many organic food products did you buy?” with a response scale from 0 (None) to 4 (More than four), while in Study 2 it was the following: “How much organic fresh fruit and vegetables have you bought during the last month?” ranging from 0 = I have not bought any organic fruit and vegetables to 4 = I have bought more than 10 kg of organic fruit and vegetables.

### Data Analysis

In order to check the adequacy of the measurement model, we conducted a confirmatory factor analysis (CFA) using the maximum likelihood method applied to covariance matrices with LISREL 8.80. Two parcels were created when the number of indicators was greater than two (i.e., in the case of attitude, intention, and trust) in order to reduce the numbers of the parameters to be estimated and to obtain conceivably smaller standard errors in the subsequent statistical analysis (Bagozzi, 1994). The measurement models of the two studies included six latent factors and twelve indicators. Goodness-of-fit was evaluated by means of the conventional indices that can be summarized as follows: χ^2^, χ^2^/df, CFI, RMSEA, and SRMR. Usually, a satisfactory model is denoted by χ^2^ not being significant, χ^2^/df ≤ 3, CFI ≥ 0.95, RMSEA ≤ 0.06, and SRMR ≤ 0.08 (Hu and Bentler, 1999). In order to estimate the reliability, the Cronbach’s alpha coefficients and composite reliabilities were determined; then, descriptive statistics were computed for all the variables (Tables 2 and 3). Finally, a structural model was used in order to test the hypothesized model of relations (Figure 1). In the next section, results from both studies will be presented in parallel.

**TABLE 2:** Descriptive statistics and reliability coefficients of trust, TPB constructs, and items—Study 1 (*n* = 237).

Constructs and items	*M*	*SD*	Cronbach’s alpha	CR
**Trust in organic food**	4.82	1.31	0.93	0.90
I perceive organic food to be reliable	4.86	1.37		
I trust in organic food products	4.79	1.38		
I trust in purchasing organic food products	4.81	1.45		
**Attitude**	5.05	1.15	0.86	0.88
Unpleasant–pleasant	5.11	1.19		
Useless–useful	4.89	1.53		
Negative–positive	5.22	1.34		
Crazy–wise	4.97	1.42		
**Subjective Norm**	4.59	1.32	0.89	0.89
Most of the people who are important to me (family, friends, acquaintances, partners) think I should/should not buy organic food products in the next month	4.72	1.28		
Most people who are important to me would like me to buy organic food products in the next month	4.47	1.49		
**PBC**	5.03	1.41	0.83	0.83
To what extent do you think buying organic food products in the next month is a behavior under your control?	5.14	1.51		
How much control do you think you have over buying organic food products in the next month?	4.92	1.55		
**Intention**	4.50	1.75	0.95	0.95
I intend to buy organic food products in the next month	4.51	1.79		
How likely is it that you will form the intention to buy organic food products in the next month?	4.57	1.83		
How likely is it that you will actually buy organic food products in the next month?	4.39	1.89		
**Behavior**	1.93	1.40	0.95	0.95
In the last month, have you personally bought organic food?	1.80	1.40		
In the last month, how many organic food products did you buy?”	2.06	1.47		

**CR, Composite Reliabilities; PBC, Perceived Behavioral Control. Every construct was scored on a 7-point response scale, except for “Behavior,” which used a 5-point one.**

**TABLE 3:** Descriptive statistics and reliability coefficients of trust, TPB constructs, and items—Study 2 (*n* = 227).

Constructs and items	*M*	*SD*	Cronbach’s alpha	CR
**Trust in organic food**	4.89	1.50	0.93	0.88
I perceive organic food to be reliable	4.99	1.56		
I trust in organic food products	4.91	1.63		
I trust in purchasing organic food products	4.76	1.60		
**Attitude**	5.13	1.38	0.91	0.93
Unpleasant–pleasant	5.11	1.19		
Useless–useful	5.08	1.52		
Negative–positive	5.24	1.48		
Crazy–wise	5.07	1.57		
**Subjective Norm**	4.91	1.40	0.92	0.92
Most of the people who are important to me (family, friends, acquaintances, partners) think I should/should not buy organic fruit and vegetables in the next month	4.98	1.41		
Most people who are important to me would like me to buy organic fruit and vegetables in the next month	4.84	1.51		
**PBC**	4.55	1.55	0.88	0.89
To what extent do you think buying organic fruit and vegetables in the next month is a behavior under your control?	4.60	1.62		
How much control do you think you have over buying organic fruit and vegetables in the next month?	4.49	1.67		
**Intention**	4.37	1.73	0.96	0.96
I intend to buy organic fruit and vegetables in the next month	4.43	1.74		
How likely is it that you will form the intention to buy organic fruit and vegetables in the next month?	4.44	1.82		
How likely is it that you will actually buy organic fruit and vegetables in the next month?	4.24	1.83		
**Behavior**	1.30	1.18	0.91	0.92
In the last month, have you personally bought organic fruit and vegetables?	1.43	1.35		
How much organic fresh fruit and vegetables have you bought during the last month?	1.18	1.09		

**CR, Composite Reliabilities; PBC, Perceived Behavioral Control. Every construct was scored on a 7-point response scale, except for “Behavior,” which used a 5-point one.**

## Results

### Descriptive Statistics

Tables 2, 3 show means and standard deviations for both the single items and the averages of composite scores of the constructs, and, in addition, reliability coefficients. Results of both studies showed that all constructs exhibited good levels of internal consistency: composite reliabilities (CRs) ranged from 0.88 to 0.96, and Cronbach’s coefficients were satisfactory. As concerns the mean scores, trust in organic food can be qualified as moderate. Altogether, participants showed a strong positive attitude toward the target behaviors and perceived them as being easy to perform. They declared only a moderate level of social pressure to execute the buying behavior and expressed a moderate intention to buy organic food or organic fruit and vegetables in the next month. On the contrary, purchasing frequency in the month before the second wave was low (Table 4): as an example 16.5% bought regularly some types of organic food and 10.1% bought regularly organic fruit and vegetables.

**TABLE 4:** Frequencies and percentages of responses on behaviors items in the two studies.

	Study 1 (*n* = 237)		
**Items**	**Response scale**	**n**	**%**
In the last month, have you personally bought organic food?	(0) No, never	57	24.1
	(1) Yes, once	48	20.3
	(2) Yes, twice	56	23.6
	(3) Yes, three times	37	15.6
	(4) Yes, regularly, every time I went shopping	39	16.5
In the last month, how many organic food products did you buy?	(0) None	57	24.1
	(1) One	28	11.8
	(2) Two	47	19.8
	(3) Three or four	55	23.2
	(4) More than four	50	21.1

	**Study 2 (*n* = 227)**		

In the last month, have you personally bought organic fruit and vegetables?	(0) No, never	77	33.9
	(1) Yes, once	54	23.8
	(2) Yes, twice	40	17.6
	(3) Yes, three times	33	14.5
	(4) Yes, regularly, every time I went shopping	23	10.1
How much organic fresh fruit and vegetables have you bought during the last month?	(0) I have not bought any organic fruit and vegetables	77	33.9
	(1) About 1 kg	69	30.4
	(2) 1–5 kg	50	22
	(3) 5–10 kg	26	11.5
	(4) I have bought more than 10 kg of organic fruit and vegetables	5	2.2

### Measurement Model

In regards to items aggregations the following parcels were created: TRUST1 was computed by averaging participants’ responses to the items “I perceive organic food to be reliable” and “I trust in purchasing organic food products”; ATT1 by averaging “unpleasant—pleasant” and “negative—positive”; ATT2 by averaging “useless—useful” and “crazy—wise”; INT2 by averaging responses to “I intend to buy organic food products/organic fruit and vegetables in the next month” and to “How likely is it that you will actually buy organic food products/organic fruit and vegetables in the next month?”. Finally for TRUST2, SNORM1, SNORM2, PBC1, PBC2, INT1, BEH1, and BEH2 the indicators used in the analyses corresponded to those observed.

According to the CFA results, the goodness-of-fit indices of the measurement model turned out to be satisfactory. In Study 1: χ^2^(39) = 54.17, *p* ≅ 0.054, χ^2^/df = 1.39, RMSEA = 0.04 [90% CI: 0.00, 0.07], CFI = 0.99, SRMR = 0.03, and the estimated factor loadings for all indicators were significant and ranged between 0.75 and 0.99 (Table 5). In Study 2: χ^2^(39) = 38.68, *p* ≅ 0.48, χ^2^/df = 0.99, RMSEA = 0.00 [90% CI: 0.00, 0.05], CFI = 1.00, SRMR = 0.02, with factor loadings that were all significant and ranging between 0.79 and 0.99. In both studies, the average variance extracted (AVE) for each construct reported in Table 5 was higher than the suggested value of 0.50 (Fornell and Larcker, 1981); furthermore, the AVE of each construct was higher than the squared correlations among the constructs, indicating good convergent and discriminant validity.

**TABLE 5:** Measurement model: Standardized factor loadings.

		Study 1 (*n* = 237)		Study 2 (*n* = 227)	
Constructs	Parcels/Items	λ _*x*_	AVE	λ _*x*_	AVE
Trust in organic food	TRUST1	0.80	0.81	0.84	0.77
	TRUST2	0.99		0.93	
Attitude	ATT1	0.94	0.79	0.92	0.87
	ATT2	0.84		0.95	
Subjective Norm	SNORM1	0.88	0.81	0.89	0.85
	SNORM2	0.92		0.95	
PBC	PBC1	0.93	0.72	0.99	0.80
	PBC2	0.75		0.79	
Intention	INT1	0.94	0.90	0.95	0.92
	INT2	0.95		0.97	
Behavior	BEH1	0.96	0.91	0.91	0.86
	BEH2	0.94		0.94	

**PBC, Perceived Behavioral Control; AVE, Average Variance Extracted.**

As concerns Study 1, the correlations among latent factors (Table 6) were all significant, except in the case of trust in organic food and PBC. The constructs that showed the highest correlation coefficient are attitude and intention (φ = 0.67). In every case, the 95% confidence intervals, which we obtained by considering two standard errors above and below the coefficients, did not include the perfect correlation (i.e., 1.00), thus supporting the fact that all measures captured distinct constructs (Bagozzi, 1994). In Study 2, the correlations among latent factors (Table 6) were all significant with the highest coefficient linking attitude and intention (φ = 0.71). Again, the 95% confidence intervals did not include the perfect correlation (i.e., 1.00).

**TABLE 6:** Correlations between latent factors.

Constructs	Trust in organic food	Attitude	Subjective norm	PBC	Intention	Behavior^a^
Trust in organic food	–	0.61 (0.05)	0.45 (0.06)	0.41 (0.06)	0.56 (0.05)	0.38 (0.06)
Attitude	0.57 (0.05)	–	0.63 (0.04)	0.42 (0.06)	0.71 (0.04)	0.49 (0.05)
Subjective norm	0.21 (0.07)	0.37 (0.06)	–	0.40 (0.06)	0.67 (0.04)	0.52 (0.05)
PBC	0.09 (0.07)^b^	0.20 (0.07)	0.24 (0.07)	–	0.53 (0.05)	0.44 (0.06)
Intention	0.44 (0.06)	0.67 (0.04)	0.51 (0.05)	0.44 (0.06)	–	0.64 (0.04)
Behavior^a^	0.32 (0.06)	0.48 (0.06)	0.41 (0.06)	0.18 (0.07)	0.58 (0.05)	–

**Standard errors in parentheses. PBC, Perceived Behavioral Control. The values of Study 1 (n = 237) are shown above the diagonal, and the ones of Study 2 (n = 227) are below the diagonal. ^*a*^In Study 1, the behavior was “to buy organic food products” and in Study 2, “to buy fresh organic fruit and vegetables.” All coefficients are significant with p < 0.01, except the one denoted by “^*b*^,” for which p = 0.18.**

### Test of the Structural Model

**FIGURE 2:**
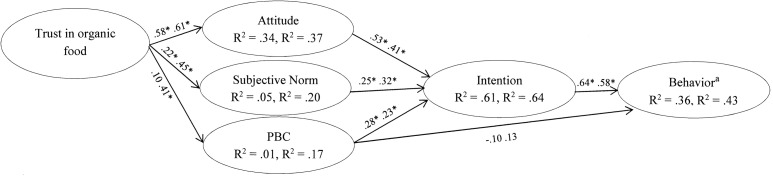
Standardized path coefficients (Study 1, *n* = 237; Study 2, *n* = 227). PBC, Perceived Behavioral Control. The first coefficients refer to Study 1 and the second ones to Study 2. *p < 0.01. ^a^In Study 1 the behavior was “to buy organic food products,” in Study 2 “to buy organic fresh fruit and vegetables.”

The overall goodness-of-fit of the model (Figure 2) was acceptable. In Study 1: χ^2^(43) = 64.93, *p* ≅ 0.01, χ^2^/df = 1.51, RMSEA = 0.05 [90% CI: 0.02, 0.07], CFI = 0.99, SRMR = 0.04; and in Study 2: χ^2^(43) = 42.60, *p* ≅ 0.49, χ^2^/df = 0.99, RMSEA = 0.00 [90% CI: 0.00, 0.04], CFI = 1.00, SRMR = 0.03.

In Study 1, the model explained 36% of the future purchase behavior variance, and only intention was significantly associated with behavior: this gave support to our first hypothesis (H1). Then, the model accounted for 61% of the variance in behavioral intention (Figure 2). Attitude showed the strongest positive association with intention, while those between subjective norm, PBC, and intention were significant but moderate. These findings supported H2, H3, and H4a and sustained the classical TPB model. PBC was not directly associated with future behavior, so H4b received no support. Trust in organic food was positively associated with attitude and subjective norm, as expected from H5 and H6. The association between trust and PBC was not significant, contrary to H7. Trust alone explained 34% of attitude variance, and 5% of subjective norm variance.

The standardized indirect effects of trust on intention, via the mediation of both attitude and subjective norm, and those of trust on purchase behavior, via the mediation of attitude, subjective norm and intention, were computed with LISREL. Results were statistically significant and equal to 0.39 (*p* < 0.001) for the indirect effect of trust on intention, and equal to 0.24 (*p* < 0.001) for the indirect effect on behavior. Moreover, intention mediated the effects of attitude (0.34, *p* < 0.001), subjective norm (0.16, *p* < 0.01), and PBC (0.18, *p* < 0.01) on purchase behavior.

In Study 2 (Figure 2), the model explained 43% of the purchase behavior variance, and only intention was significantly associated with this behavior, supporting our first hypothesis (H1). The model accounted for 64% of the variance in behavioral intention; attitude and subjective norm showed the strongest positive associations with intention. In addition, the association between PBC and intention was significant. These findings supported H2, H3, and H4a and, consequently, the classical TPB model. For a second time, in contrary to H4b, PBC was not directly associated with future behavior. Trust in organic food was positively associated with attitude, subjective norm, and PBC, as predicted by H5, H6, and H7. Again, trust alone explained a good portion of attitude variance (37%) and smaller quotas of subjective norm and PBC variances. The analysis of the indirect effects showed that the standardized indirect effect of trust on intention via the mediation of attitude, subjective norm, and PBC was significant (0.49, *p* < 0.001); the indirect effect of trust via the mediation of attitude, subjective norm, PBC, and intention on behavior turned out to be significant (0.34, *p* < 0.001). In addition, in this case, intention mediated the effects of attitude (0.24, *p* < 0.001), subjective norm (0.19, *p* < 0.01), and PBC (0.14, *p* < 0.01) on purchase behavior. In both studies, the modification indices (MI) concerning the direct paths between trust and intentions and between trust and behaviors were lower than 3.84 indicating a no significant improvement in model fit as a result of freeing these parameters (Bagozzi, 1994); therefore, we concluded that the effects of trust were completely mediated by the TPB constructs.

## Discussion

The persistent increase in organic food sales in the last decades has definitely attested both the growing interest and trust that consumers have in a food category consisting of products that are supposed to have been grown naturally, without the use of any kind of chemicals. Conversely, little research seems to have investigated the possible relationship between trust in organic food and the antecedents of both buying intention and the actual purchase, following one of the most important socio-psychological theoretical frameworks, the TPB.

Since consumers are not in a position to have access to complete information or control over the overall process of organic food production and sales, trust must necessarily play a role in the decision-making process when people assume that food labeled as “organic” is safe, healthy, natural, and tasty, and that its consumption also has environmental benefits. In fact, several studies carried out in the USA and Europe showed that beliefs regarding organic food characteristics were related to taste and healthiness, as well as to the perceived benefits for the environment and animal welfare (e.g., Saba and Messina, 2003; Arvola et al., 2008; Zagata, 2012).

Findings of the two studies presented in this paper highlighted that the TPB model has a strong explanatory value. In fact, the hypotheses concerning the relations between attitudes, subjective norm, PBC, and intentions received support (i.e., H2, H3, and H4a). Attitudes toward the behaviors had the strongest effects on intentions; subjective norm and PBC had significant but comparatively lower effects on them. Altogether, the results suggest that a positive attitude toward the purchase of organic food in general, or fresh organic fruit and vegetables in particular, predicts intentions and, indirectly, actual behaviors, and this is consistent with previous findings in the literature (e.g., Aertsens et al., 2009; Scalco et al., 2017; Carfora et al., 2019). Moreover, in line with the literature (Armitage and Conner, 2001), subjective norm was shown to have the weakest impact on intention compared to the other TPB components. However, the positive effect of subjective norm indicates that expectations about food purchases shared with important others, such as family members and friends, positively affect the willingness of consumers to buy organic food.

In regards to PBC, it seemed to play a minor role than attitude in intention formation. Again, this is in line with the meta-analysis by Scalco et al. (2017), but it diverges from results of studies conducted in the Italian context, which proposed increasing the consumption of organic food by increasing its availability and, consequently, the perceived control of customers (e.g., Giampietri et al., 2018; Carfora et al., 2019).

Intentions predicted self-reported behavior over 1 month, supporting H1. Instead, H4b on the direct effect of PBC on behavior was not confirmed. Following Ajzen (1991), we could deduce that our participants considered the proposed behaviors as completely under their volitional control. However, since measures of actual control were not available—as they are not for most behaviors (Fishbein and Ajzen, 2010)—we should be aware of the fact that PBC could also not to be the best proxy of actual control in this behavioral domain. Future appropriately designed studied could deal with this issue.

H5 received support since trust in organic food was positively associated with attitude in both studies, mirroring the extant literature (e.g., Teng and Wang, 2015; Ricci et al., 2018), and it explained about a third of the attitude variance. Trust was also positively associated with subjective norm and, in the case of Study 2 only, with PBC, thus offering support to H6 and, partially, to H7. Besides, trust explained lower quotas of subjective norm and PBC variances, compared to attitude.

Consumer trust plays a key role in developing an overall positive evaluation toward organic food. Its effect on attitudes showed that the more consumers trusted organic food, the more they showed positive attitudes toward it. The influence of trust on subjective norm told us that the higher the trust score was (and, consequently, the more our participant accepted being vulnerable to possible misconducts or frauds), the more they relied on opinions of their important referents. Indeed, as mentioned before, it is reasonable to assume that many consumers do not have sufficient information on organic food production and manufacturing and, in some sense, when they decide to buy it, their judgment is necessarily sensitive to those of others.

The effect of trust on PBC was significant only in Study 2. This finding suggests that trust in organic food could act as a facilitator for consumers’ behavior, while a lack of trust could act as an obstacle to it, as argued by Hansen et al. (2018) in their study on trust in social networking services.

H8 and H9 were supported by the data. Trust in organic food was indirectly associated with intentions and behaviors via its significant effects on attitudes and subjective norm (and on PBC, but only in Study 2). In our view, individual differences in terms of trust could have an indirect effect on intentions to buy organic food in general, and fresh organic fruit and vegetables in particular, and on actual purchase behaviors, and this hypothesis should be explored further in future studies.

The hypothesized model, which incorporated trust as a background variable in the TPB framework, yielded a robust performance with regard to its explanatory power. In Studies 1 and 2, it explained 61% and 64% of intention variance, respectively. These quotas are similar to those reported by Carfora et al. (2019) and slightly lower than that published by Qi and Ploeger (2019). As concerns future behavior, the model explained 36% and 43% of variance; once more, these quotas are in accordance with Carfora et al. (2019). Consistent with findings in other behavioral domains, the lowest quotas of explained behavior variance compared to those of intentions may be due to several factors, such as: (a) issues regarding the validity of self-reported behavior measures, (b) events that occurred between the assessment of intentions and behaviors, which may have produced changes in intentions, and (c) unanticipated obstacles that may have prevented the individuals from carrying out their intentions (de Leeuw et al., 2015).

Finally, TPB-based processes are similar in the case of a general target behavior (the purchase of organic food) and of a specific behavior (the purchase of fresh fruit and vegetables), and we see this as promising in terms of the possible generalizability of results in future replicas with different targets.

Turning to the potential practical implications of our research, we argue that the findings may be valuable for different stakeholders, for instance, practitioners, marketers, policymakers, and even firms interested in the organic food industry, and for several reasons. First, intention emerged as the only significant predictor of purchase behavior: namely, consumers seemed to buy organic food because they had planned to do it, and our findings suggested that buying intentions are boosted mainly by attitude, subjective norm and perceived control, but also indirectly by trust. However, both the frequency and quantity of self-reported purchase behaviors were low, and therefore, there is still room for interventions aimed at increasing organic consumption and strengthening the demand of these products. Second, although attitudes and PBC are already quite positive, public policy initiatives should try to improve the perceived value of organic food products for the individual and for society as a whole. Additionally, marketers should design marketing campaigns focused on the personal advantages of organic food consumption, such as health benefits (Kushwah et al., 2019), and on facilitating the perception of control, thus supporting individuals in overcoming obstacles and barriers (e.g., the cost of sustainable products and the difficulty of finding them in stores).

Our results also indicate that family, relatives, and friends could contribute to shaping individual intentions to buy organic food and to strengthening green or sustainable purchase practices. In this case, public policy initiatives should be directed at the interdependent nature of family and community relationships, stressing both the ethical value of organic food purchases and the societal or altruistic value of organic food products and consumption (e.g., environmental, animal, and farmers’ welfare). Third, positive attitudes toward the purchase of organic food, subjective norm and also PBC can be increased if buyers consider organic food to be trustworthy. In this case, marketers could use this evidence to build communication campaigns intended to promote trust in these products, especially in non-buyers.

As we stated in the Introduction, it is important to remember that trust may serve as a “shortcut” when, as in the case of organic food, consumers have limited information about and exposure to the production or preparation of these products, and when direct relationships with food producers are rare (Hartmann et al., 2015). Thus, it may be the case that consumers perceive an organic label as a symbol of quality *per se* and as a strong heuristic cue (Vega-Zamora et al., 2014). Since any kind of organic food can be seen as reliable, consumers’ trust in organic food may be largely based only on the intrinsic value of the “organic” label (Ayyub et al., 2018). The increasing availability of products labeled as “organic,” without all the appropriate information being shared with customers, or without a parallel increase in consumers’ awareness on how organic food should be produced or manufactured, may potentially enhance the risk of fraud in this sector, given the “brand” value that the word “organic” has assumed over recent years. Since the use of shortcuts in decision-making can sometimes be risky, consumers should be invited to increase their knowledge about this food category and to ask for transparent information regarding constituents, quality, and controls carried out in the organic food sector. Future studies should be devoted to these issues.

Our studies can offer a significant contribution to the emerging literature on the purchasing of organic food in various ways. First, antecedents of consumers’ decisions to buy organic food, following a renowned socio-psychological approach, that is, the TPB, were explored in detail. Second, it presented an initial comparison between different types of organic food purchasing, something that is rarely investigated. Third, to our knowledge, it is one of the few studies that has considered a prospective purchase. Indeed, most recent claims in the literature have advocated the need to focus on actual choice behavior along with behavioral intentions because behavioral intentions alone may not represent actual purchase behavior accurately (Yadav and Pathak, 2016a).

Despite these points of strength, there are some limitations that must be acknowledged. First, we used two convenience samples, and thus, generalizability to the entire population is questionable. Behavior was measured through self-report items, which could be subject to social desirability or social approval biases, and to retrieval inaccuracy. Moreover, our study, although it considers a prospective measure of behavior, is cross-sectional in design, and therefore, it does not allow the assessment of proper causal relations. Finally, socio-demographic variables were not considered in our models even though numerous other background factors, such as education, income, and the area of residence (town or rural), could also be associated with the constructs and relationships considered in our studies.

Future studies should use the same extended TPB model in order to predict consumers’ purchase intentions regarding other specific organic products, such as processed fruit or bakery goods, personal hygiene, clothes, and furniture. Finally, in our studies, as in the majority of research inspired by the TPB, beliefs associated with attitude toward the purchase behaviors (i.e., behavioral beliefs), subjective norm (i.e., normative beliefs), and PBC (i.e., control beliefs) were not assessed. Instead, in order to project interventions designed to encourage the purchase of organic food, the knowledge of the specific beliefs underlying attitude, subjective norm and PBC would provide useful information. Future studies should also examine antecedents of trust (like information or exposure to the production processes of organic products) in order to fully understand their role in decision-making and developing tailored interventions.

## Conclusion

The results of the present study confirm the efficacy of the TPB as a framework for understanding intentions and behavior in the field of organic food purchasing, and the current test significantly contributes to the body of evidence for the predictions specified in the model. Consumer trust had significant effects on organic food purchasing via the antecedent variables of the TPB, and it turned out to be crucial for promoting intentional behaviors. Overall, our results are in line with Fishbein and Ajzen (2010), who claimed that the TPB allows the incorporation of various background factors and the testing of the mediating influence of these factors on intentions and behavior. However, given that trust may be a risky “shortcut” in decision-making processes, consumers should become more informed about organic products and claim the right to have transparent information regarding their quality. Producers and retailers, for their part, should promote communication campaigns and solid relationships with consumers in order to build knowledge and loyalty between all the different actors involved in the manufacturing, processing, and selling of organic food.

## Data Availability Statement

The raw data supporting the conclusions of this article will be made available by the authors, without undue reservation, to any qualified researcher.

## Ethics Statement

The studies involving human participants were reviewed and approved by Ethical Committee of the School of Psychology at Padua University (code 1529). The patients/participants provided their written informed consent to participate in this study.

## Author Contributions

LC and AM designed the studies, supervised the data collection, and contributed to the writing of the manuscript. LC performed data analysis. AB contributed to the data analysis and to the writing of the manuscript. AM supervised the research project. All authors revised and approved the submitted version.

## Conflict of Interest

The authors declare that the research was conducted in the absence of any commercial or financial relationships that could be construed as a potential conflict of interest.
